# P-73. Pyogenic And Brucellar Spondylodiscitis: Comparative Analysis Of Clinical, Laboratory, And Evolutionary Features

**DOI:** 10.1093/ofid/ofae631.280

**Published:** 2025-01-29

**Authors:** Fatma Hammami, Khaoula Rekik, Fatma Smaoui, Amal Chakroun, Chakib Marrakchi, Makram Koubaa, Mounir Ben Jemaa

**Affiliations:** Infectious Diseases Department, Hedi Chaker University Hospital, University of Sfax, Tunisia, Sfax, Sfax, Tunisia; Infectious Diseases Department, Hedi Chaker University Hospital, University of Sfax, Tunisia, Sfax, Sfax, Tunisia; Infectious Diseases Department, Hedi Chaker University Hospital, University of Sfax, Tunisia, Sfax, Sfax, Tunisia; Infectious Diseases Department, Hedi Chaker University Hospital, University of Sfax, Tunisia, Sfax, Sfax, Tunisia; Infectious Diseases Department, Hedi Chaker University Hospital, University of Sfax, Tunisia, Sfax, Sfax, Tunisia; Infectious Diseases Department, Hedi Chaker University Hospital, University of Sfax, Tunisia, Sfax, Sfax, Tunisia; Infectious Diseases Department, Hedi Chaker University Hospital, University of Sfax, Tunisia, Sfax, Sfax, Tunisia

## Abstract

**Background:**

Spondylodiscitis is an infection of the vertebral and the intervertebral disc. The clinical presentation varies according to the underlying etiology. We aimed to compare the clinical, laboratory and evolutionary features between pyogenic and brucellar spondylodiscitis.

**Methods:**

We conducted a retrospective study including patients hospitalized in the infectious disease department for pyogenic or brucellar spondylodiscitis between 1998 and 2022.

**Results:**

We encountered 44 cases of brucellar spondylodiscitis (BS) (50.6%) and 43 cases of pyogenic spondylodiscitis (PS) (49.4%). Patients with PS were significantly older (60±16 years vs 50±16 years; p=0.007). A rural origin was significantly more frequent among BS patients (88.6% vs 55.8%; p=0.001). While motor deficit was significantly more frequent among PS patients (33.3% vs 11.4%; p=0.014), nights sweats were significantly more frequent among BS patients (72.7% vs 25.6%; p< 0.001). As to gender, fever and back pain, no significant difference was noted. The lumbar spine was the most affected site among BS group (74.4%) and PS group (72.1%), with no significant difference (p=0.8). Laboratory investigations including white blood cells count (11153±4952/mm^3^ vs 6719±2910/mm^3^; p< 0.001), erythrocyte sedimentation rate (84±31mm/h vs 50±33mm/h; p< 0.001) and C-reactive protein levels (108±87mg/l vs 32±28mg/l; p< 0.001) were significantly higher among PS group. Surgical treatment was indicated in 9.3% of PS cases and in 2.3% of BS cases, with no significant difference (p=0.3). Two patients with PS were dead (4.6%). Sequelae were noted among PS cases (41.9%) and BS cases (38.6%), with no significant difference (p=0.7).

**Conclusion:**

Although PS patients were older, presented more frequently with motor deficit and had higher inflammatory markers, the disease evolution was similar in comparison with BS patients.

**Disclosures:**

**All Authors**: No reported disclosures

